# Burkitt's Lymphoma Presented as Advanced Ovarian Cancer without Evidence of Lymphadenopathy: CT and MRI Findings

**DOI:** 10.1155/2013/940160

**Published:** 2013-03-30

**Authors:** Lucia Manganaro, Silvia Bernardo, Maria Eleonora Sergi, Paolo Sollazzo, Valeria Vinci, Alessandra De Grazia, Anna Clerico, Maria Giovanna Mollace, Matteo Saldari

**Affiliations:** ^1^Department of Radiological Oncological and Anatomopathological Sciences, Umberto I Hospital, “La Sapienza” University of Rome, Viale Regina Elena 324, 00161 Rome, Italy; ^2^Department of Pediatric Oncology, Umberto I Hospital, “La Sapienza” University of Rome, Viale Regina Elena 324, 00161 Rome, Italy

## Abstract

Burkitt's lymphoma is a rare non-Hodgkin's lymphoma which can occasionally involve the ovary and may cause confusion for the clinician since its presentation might mimic other much more frequent tumors. We present a case of a 23-year-old woman with sporadic Burkitt's lymphoma presented as advanced ovarian cancer with bilateral ovarian masses, peritoneal carcinomatosis, ascites, and marked elevation of CA-125. Liver involvement and atypical bone lesions, such as the cranial vault and the iliac wing, were also detected without evidence of lymphadenopathy. We describe the MRI and CT findings of simultaneous ovarian and bone lesions, which have never been reported in literature in a patient with Burkitt's lymphoma, before and after one cycle of chemotherapy. In evaluating any ovarian neoplasm in a young woman, Burkitt's lymphoma should be considered as a possibility, particularly if associated with bone lesions. MRI is the most useful tool to characterize the ovarian lesions and suggest the diagnosis before the histopathological results.

## 1. Introduction

The updated WHO Classification of Lymphoid Neoplasms (2008) identifies Burkitt's lymphoma (BL) as a highly aggressive and fast growing mature B-cell neoplasm that often presents in extranodal sites or as an acute leukemia. Although the incidence of BL is low, accounting for only 1~2% of all lymphomas in western countries, it is one of the most common types of malignant tumors in children (endemic, sporadic) and immunocompromised hosts [1]. Involvement of the ovary by BL can be a manifestation of a systemic disease or, even more rarely, true primary ovarian lymphoma [2]. We present here the MRI and CT findings of a case of sporadic Burkitt's lymphoma mimicking an ovarian cancer with associated bone involvement but without evidence of lymphadenopathy. A follow-up CT scan after 4 weeks, following one cycle of chemotherapy, was performed and showed a remarkable reduction of the ovarian lesions but not of the bone ones.

## 2. Case Report

**Figure 1:**
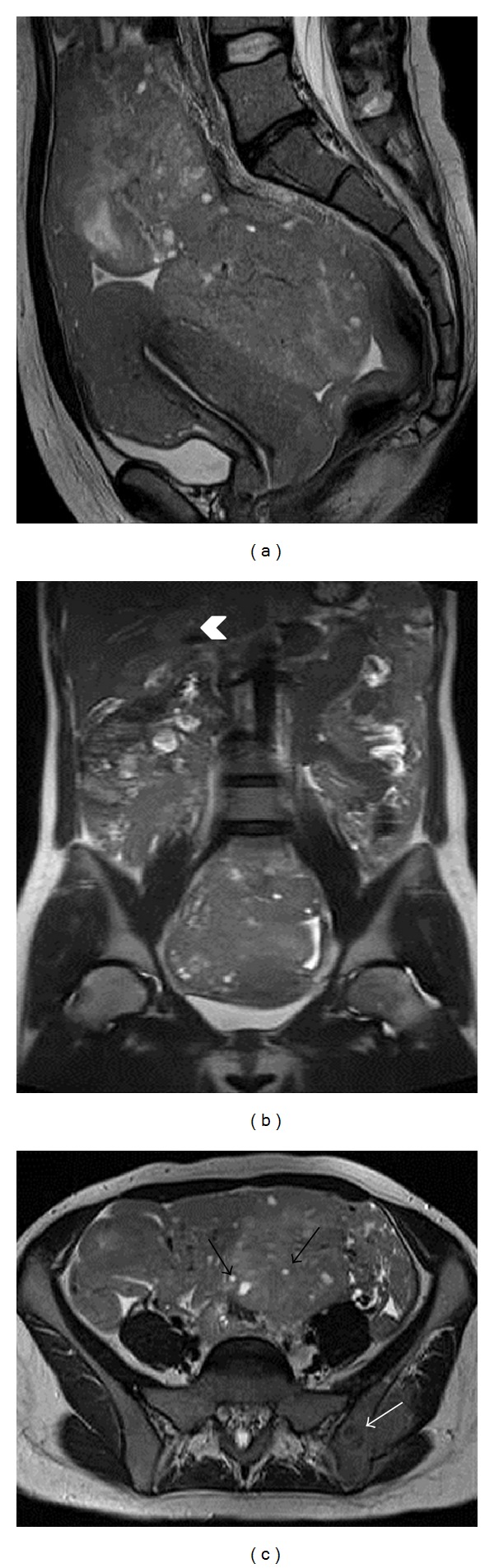
Burkitt's lymphoma presented as two well-encapsulated pelvic masses of intermediate signal intensity on sagittal (a) T2-weighted MR image. Coronal (b) T2-weighted image shows also liver involvement (arrowhead). A bone lesion in the left iliac wing (white arrow) and round cysts of high signal intensity both within and at the periphery of the ovarian lesions (black arrows) are seen on an axial (c) T2-weighted image.

We present a case of a 23-year-old nulliparous woman who was admitted to the hospital because of a two-month facial pain, increased abdominal circumference with constipation, pain to her lower left arm, excessive sweating, and weight loss. Abdominal ultrasound showed a large pelvic mass measuring 13 cm in diameter and a small amount of free fluid in the pelvis. A nodular hypoechoic area in segment IV of the liver was also detected. Tumoral markers were normal, with the exception of CA-125 which was 343 U/mL (normal limits < 30 U/mL). Blood count revealed WBC 13.38 × 10^3^/mcL, neutrophils 8.7 × 10^3^/mcL, monocytes 1.6 × 10^3^/mcL, and lymphocytes 2.9 × 10^3^/mcL. Laboratory tests gave the following values: LDH 1774 U/L, ALT 239 U/L, AST 189 U/L, gamma GT 108 U/L, and uric acid 12.7 mg/dL. A pelvic MRI examination with a 1.5 T unit was performed to further characterize the lesions. Multiplanar (axial, coronal, and sagittal) conventional T1- and T2-weighted and DWI images were acquired. The exam showed two well-encapsulated masses with predominantly solid appearance, the maximum total size of about 18 cm along the latero-lateral diameter and 7 cm along the anteroposterior diameter, localized in the pouch of Douglas and in the left hypogastrium (Figure 1). The masses presented homogeneously low signal intensity on T1-weighted images and intermediate signal intensity on T2-weighted images. Small round cysts of high signal intensity on T2-weighted images were seen both within and at the periphery of the lesions. MR examination showed some bone lesions localized in the left iliac wing, left femoral head and neck, anterior-superior iliac spine, and acetabulum. Involvement of the liver hilum was also observed.

**Figure 2:**
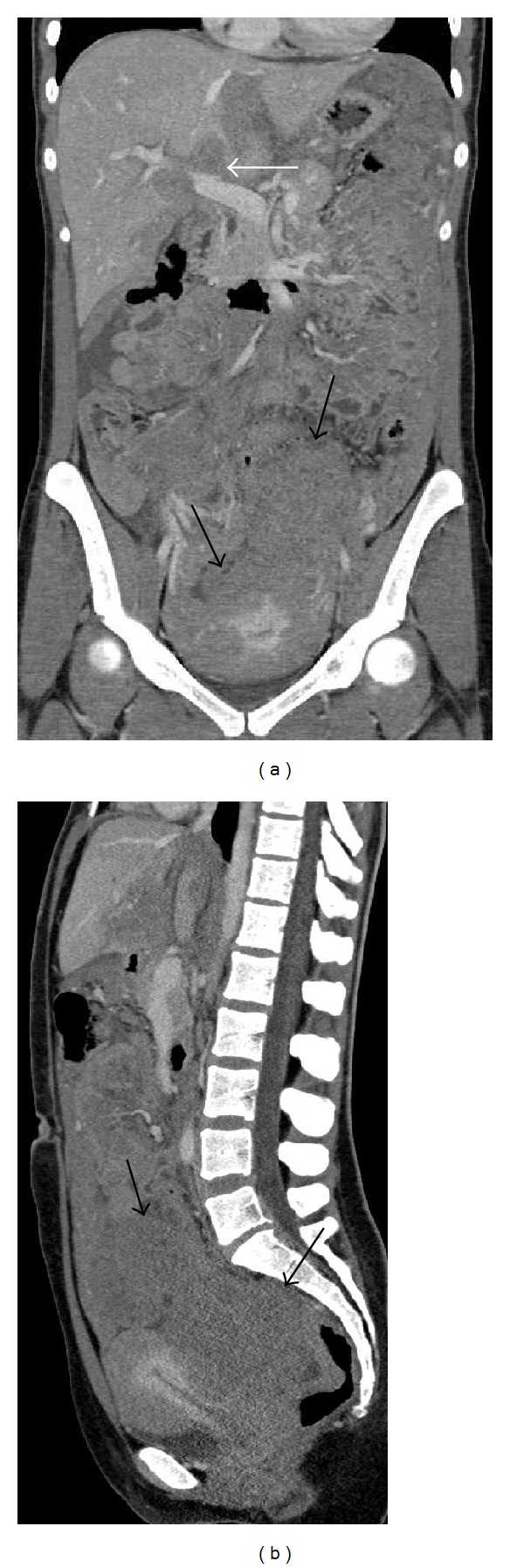
Coronal (a) and sagittal (b) CT images show two hypodense masses (black arrows), with relative structural homogeneity and without significant necrosis, hemorrhage, or calcifications. Note that there are also secondary lesions affecting the liver hilum (white arrow).

**Figure 3:**
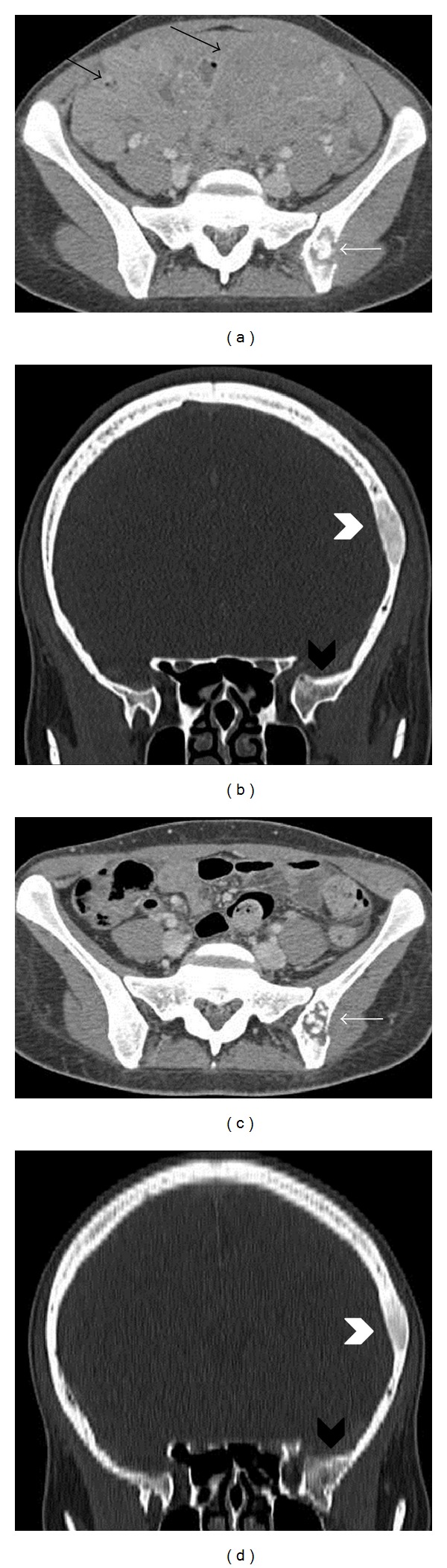
Axial CT image (a) shows two pelvic masses (black arrows) with mild contrast enhancement and a bone lesion in the left iliac wing (white arrow). There is also a lesion in the left parietal bone (white arrowhead) and in the left greater sphenoid wing (black arrowhead) without involvement of the cerebral parenchyma (b). Axial CT image of the pelvis (c) and coronal CT image of the skull (d) do not show any reduction in size of the parietal (white arrowhead), sphenoid (black arrowhead), and iliac (white arrow) bone lesions following one cycle of chemotherapy.

At this point we performed a full-body CT scan for the staging of the tumor. The exam showed the intraperitoneal pelvic masses seen on MRI and involving both ovaries (Figure 2). The lesions were hypodense, with relative structural homogeneity, mild contrast enhancement and without significant necrosis, hemorrhage, or calcifications. Diffuse parietal thickening of the intestinal loops and multiple secondary lesions affecting the liver hilum with infiltration of the hepatic parenchyma were also revealed. Osteoblastic lesions were identified in the left parietal bone, in the left greater sphenoid wing, and in the left iliac wing (Figure 3). 

A bone scintigraphy showed all the bone lesions seen at MRI and CT as some hyperfixation areas of the osteotropic tracer (Tc99m-MDP).

Liver biopsy examination made histological diagnosis of diffuse non-Hodgkin lymphoma with morphological and immunohistochemical features of Burkitt's lymphoma (sporadic). Neoplastic cells were positive for CD20, CD19, CD10, Bcl-6, and MUM-1 and negative for TdT, CD30, CD3, and cytokeratin (clone MNF 116), and the proliferative index assessed by Ki-67 was 100%. A bone marrow aspiration was performed and showed the presence of a hypercellular marrow with 99% of blasts ranging from medium to large size characterized by a basophilic and markedly vacuolated cytoplasm. A lumbar puncture was performed, and it was “negative for malignant cells.”

The patient was treated with chemotherapy according to the protocol of AIEOP LNH-97 R4 for B-cell non-Hodgkin's lymphoma and B-ALL (stage IV with LDH ≥ 1000 U/L and negative CNS) and presented considerable clinical improvement after one week of treatment. 

**Figure 4:**
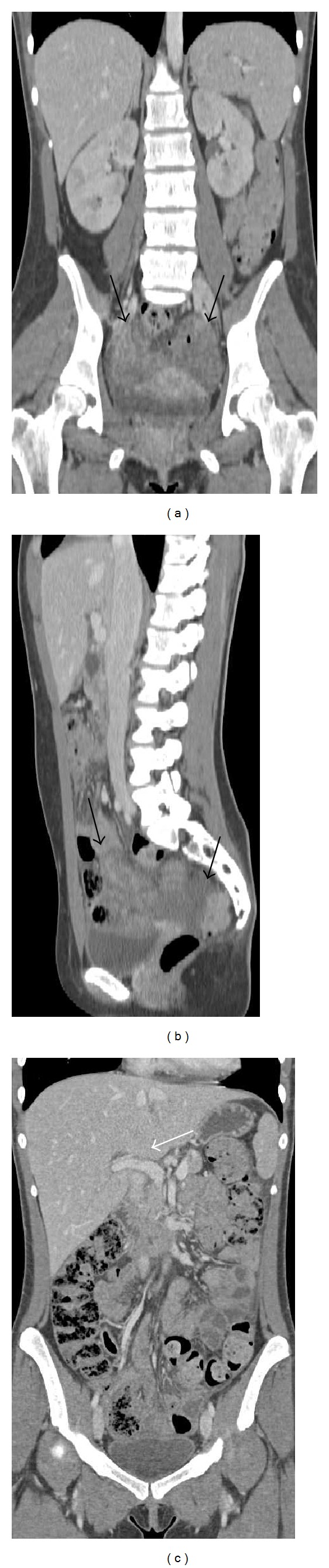
Coronal ((a) and (c)) and sagittal (b) CT images show a remarkable reduction of the ovarian masses (black arrows) and total regression of the neoplastic tissue which infiltrated the liver parenchyma (white arrow), following one cycle of chemotherapy.

A follow-up CT scan at 4 weeks showed a remarkable reduction of the adnexal masses and total regression of the neoplastic tissue which infiltrated the liver parenchyma and of the peritoneal carcinosis (Figure 4). However, no significant change in size of the bone lesions was reported, and all the bone formations showed a change of the density with an osteolitic pattern (Figure 3).

## 3. Discussion

Lymphomatous processes in female genital tract are rare although the ovaries are the most common site to be involved [2]. Involvement of the ovary can be primary or secondary, but the occurrence of lymphomas primarily arising in the ovaries has long been debated because there is no lymphoid tissue within the ovary. Most authors agree that this is the first manifestation of an occult nodal disease or a secondary localization due to systemic diffusion of a lymphomatous process [3]. The majority of lymphomas involving the ovary are of B-cell phenotype; among these, Burkitt's lymphoma and diffuse large B-cell lymphoma (DLBCL) are the most common types [4]. 

Diagnosis of intra-abdominal Burkitt's lymphoma is often delayed, as symptoms could be nonspecific or even absent. Abdominal pain, distension, nausea, vomiting, amenorrhea, irregular menses, and osteoarticular aches are the most common findings [3]. After the abdomen, the next most common site of presentation is the head and neck, and majority of BL patients (70%) present with advanced stages [1]. Some cases are characterised by extensive marrow infiltration with possible bone pain as a presenting feature. Although BL frequently affects the maxilla and mandible, other more rare bone sites, such as the iliac wing, the central skull base, and the cranial vault, could be involved [5], as in our patient. To our knowledge this is the first case to report imaging findings of Burkitt's lymphoma with ovarian and skeletal involvement without evidence of lymphadenopathy. 

At CT ovarian lymphoma presents as hypodense lesions with mild contrast enhancement, without significant necrosis, hemorrhage, or calcifications and with relative structural homogeneity [6, 7]. 

Although CT scan remains the most important examination for staging and followup after chemotherapy [6], MRI provides a better characterization than CT and is more useful in distinguishing solid from complex cystic fluid components and assessing spatial relations of pelvic masses. 

Ferrozzi et al. [8] described the MRI findings of five patients with ovarian non-Hodgkin lymphoma. MRI showed the ovarian lesions as homogenous masses, round or oval in morphology, that exhibited homogeneously low signal intensity on T1-weighted images and intermediate to high intensity on T2-weighted images. 

Mitsumori et al. [9] and Crawshaw et al. [10] presented two cases of primary ovarian Burkitt's type NHL. At MRI the masses were of low signal intensity on T1-weighted images and of intermediate signal intensity on T2-weighted images with well-defined, round, and high signal intensity cysts at the periphery, consistent with follicles. In our case the cysts were also localized within the lesion and not only at the periphery.

Other neoplasms should be differentiated from NHL involving the ovary such as granulocytic sarcoma, dysgerminoma, adult granulosa cell tumor, and fibrothecoma. Granulocytic sarcoma presents intermediate signal intensity on T1-weighted images and hypointensity on T2-weighted images and is less likely to be bilateral [11]. In our case, also a diagnosis of fibroma or thecoma could be excluded because there was no fibrous component on MRI, and these tumors have low signal intensity on T1-weighted MRI images and very low signal intensity on T2-weighted images. Other tumors often appear with radiological evidence of hemorrhage or necrosis, which were absent in our patient, such as granulosa cell tumor and dysgerminoma. Bilateral ovarian lymphomas should also be differentiated from metastatic involvement of the ovaries. Ovarian metastases represent ~30% of all ovarian neoplasms and are generally bilateral with mixed appearance (solid and cystic) [12]. Finally epithelial tumors are typically primarily cystic although associated with varying proportions of solid tissue. As in our patient, BL associated with peritoneal carcinomatosis, ascites, and high serum levels of Ca125 was detected in some cases [10], mimicking an ovarian cancer and causing confusion for the clinician. However, bone involvement from ovarian malignancies is relatively rare whereas it is more common in cases of NHL. Bone lesions associated with a homogenous and well-defined ovarian neoplasm, which doesn't show an infiltrative pattern of growth or regressive changes, in a young woman, could be suggestive of lymphoma. The MRI is the most useful and comprehensive tool to characterize ovarian lesions and suggest the diagnosis before the histopathological results.
